# 非小细胞肺癌合并Trousseau综合征以急性脑梗死为表现的临床及影像学特征

**DOI:** 10.3779/j.issn.1009-3419.2021.102.01

**Published:** 2021-01-20

**Authors:** 腾飞 袁, 俊萍 汪

**Affiliations:** 300052 天津，天津医科大学总医院放射科，天津市功能成像重点实验室 Department of Radiology, Tianjin Key Laboratory of Functional Imaging, Tianjin Medical University General Hospital, Tianjin 300052, China

**Keywords:** Trousseau综合征, 肺肿瘤, 脑梗死, 高凝状态, 磁共振, 扩散加权成像, Trousseau syndrome, Lung neoplasms, Cerebral infarction, Hypercoagulability, Magnetic resonance, Diffusion-weighted imaging

## Abstract

**背景与目的:**

急性脑梗死是Trousseau综合征（Trousseau syndrome, TS）的一种表现形式，但相对少见，往往容易被临床医师忽视。本研究探讨非小细胞肺癌（non-small cell lung cancer, NSCLC）合并TS以急性脑梗死为表现的临床、实验室检查及影像学特点。

**方法:**

回顾性收集25例以急性脑梗死为表现的NSCLC合并TS患者的临床资料、实验室检查和影像学资料，进行分析总结。

**结果:**

25例患者中男性18例，女性7例，年龄39岁-78岁，其中腺癌22例、鳞癌2例和大细胞癌1例；所有患者均有急性脑梗死的临床症状和体征；血浆D-二聚体明显升高，凝血酶原时间及活化部分凝血活酶时间均有不同程度的缩短；所有患者在头部磁共振成像（magnetic resonance imaging, MRI）[扩散加权成像（diffusion-weighted imaging, DWI）序列]平扫上均表现为累及多个颅内动脉供血区的急性多发性脑梗死灶，头磁共振血管成像（MR angiography, MRA）上梗死灶对应的供血血管管腔未见中重度狭窄。

**结论:**

NSCLC合并急性多发性脑梗死是TS的少见表现类型，其特点是累及多个动脉供血区的急性多发性脑梗死灶伴有明显高凝状态；提高对该病的早期认识可以为临床诊疗提供一定的帮助。

## Abstract (en)

**Background and objective:**

Acute cerebral infarction is a form of Trousseau syndrome (TS), but is relatively rare and often overlooked by clinicians. The aim of this study was to investigate the clinical, laboratory and imaging features of acute cerebral infarction in non-small cell lung cancer (NSCLC) patients with TS.

**Methods:**

Clinical data, laboratory examination and imaging data of 25 NSCLC patients with TS presented with acute cerebral infarction were collected retrospectively for analysis.

**Results:**

Of the 25 patients, 18 males and 7 females, aged 39-78 years old, including 22 cases of adenocarcinoma, 2 cases of squamous cell carcinoma, and 1 case of large cell carcinoma; all patients had clinical symptoms and signs of acute cerebral infarction; plasma D-dimer was significantly increased, and prothrombin time and activated partial thromboplastin time were shortened to varying degrees; all patients showed acute multiple cerebral infarction foci involving multiple intracranial arterial blood supply areas on plain head magnetic resonance imaging (MRI) [diffusion-weighted imaging (DWI) sequence], the blood supply vessel lumen corresponding to the infarction foci did not show moderate to severe stenosis on the head MR angiography (MRA).

**Conclusion:**

NSCLC with multiple acute cerebral infarctions is a rare manifestation of TS, which is characterized by multiple acute cerebral infarctions involving multiple arterial blood supply areas with significant hypercoagulability. Improving the early understanding of this disease can provide some help for clinical diagnosis and treatment.

随着外科手术、放射治疗、靶向药物和免疫治疗的发展，非小细胞肺癌（non-small cell lung cancer, NSCLC）患者的生存期不断延长，但血栓栓塞事件发生率却明显升高，成为患者第二大常见死亡原因^[1]^。1895年法国医生Armand Trousseau首次报道了恶性肿瘤与浅表性游走性血栓静脉炎的相关性，之后将恶性肿瘤患者因凝血和纤溶机制异常而导致的各种血栓栓塞事件统称为Trousseau综合征（Trousseau syndrome, TS）^[2]^。TS的临床表现包括深静脉血栓形成（deep vein thrombosis, DVT）、肺栓塞（pulmonary embolism, PE）与非细菌性血栓性心内膜炎（non-bacterial thrombotic endocarditis, NBTE）相关的慢性弥散性血管内凝血（disseminated intravascular coagulation, DIC）和动脉血栓形成^[3]^。急性脑梗死是TS的一种表现形式，但相对少见，往往容易被临床忽视。本研究通过分析25例NSCLC患者合并TS以急性脑梗死为表现的临床及影像学特征，以提高对它的认识，为临床诊疗提供一定的帮助。

## 1 资料与方法

### 1.1 临床资料

回顾性搜集自2014年12月-2020年9月于天津医科大学总医院临床诊断为NSCLC患者合并TS以急性脑梗死为表现的患者25例，其中男18例，女7例，年龄39岁-78岁，平均年龄（56±11）岁。纳入标准：①病理诊断为NSCLC；②有急性脑梗死的临床症状和体征；③符合全国第四届脑血管病学术会议制定的脑梗死诊断标准，并结合头颅磁共振成像（magnetic resonance imaging, MRI）平扫[扩散加权成像（diffusion-weighted imaging, DWI）是目前诊断急性脑梗塞最敏感的序列，明显优于其他常规MRI序列（T1WI和T2WI）]和增强检查结果，DWI上呈高信号，表观弥散系数（apparent diffusion coefficient, ADC）值降低，无强化，影像学诊断为急性脑梗死；④TOAST分型为原因不明性脑卒中，经两位神经内科和两位医学影像科主治医师讨论一致考虑为TS合并急性脑梗死。排除标准：①合并血液系统肿瘤、其他原发性实性肿瘤或颅内转移瘤；②心力衰竭（心脏彩超提示射血分数 < 40%）；③低血压：肱动脉血压 < 90 mmHg/60 mmHg；④重度贫血：血红蛋白（hemoglobin, Hb） < 60 g/L；⑤心脏彩超及心电图发现有可能形成心脏栓子的疾病：房颤、扩张型心肌病、风湿性心脏病、感染性心内膜炎、心瓣膜病、急性心肌梗死、心房黏液瘤等；⑥患者临床资料不完整，包括详细的病史、实验室检查、影像学及病理学检查。纳入和排除标准详见图 1。

**图 1:**
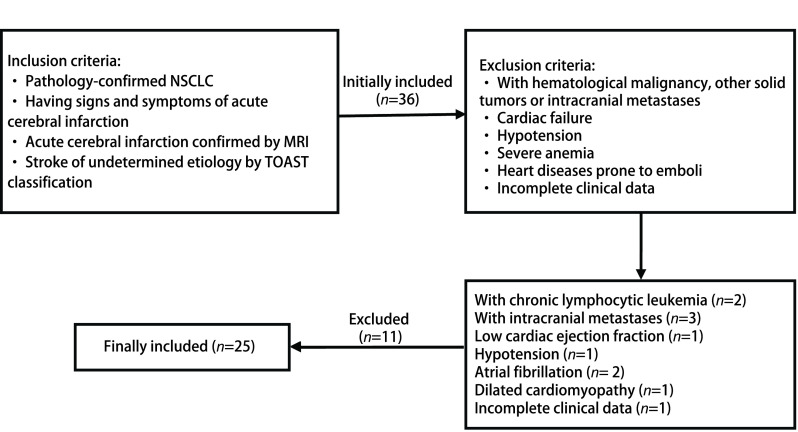
纳入和排除标准 Inclusion criteria and exclusion criteria. NSCLC: non-small cell lung cancer; MRI: magnetic resonance imaging.

### 1.2 临床特征及实验室检查分析

收集患者以下临床资料：①有无传统缺血性脑卒中（动脉粥样硬化）危险因素，如高血压、糖尿病、高血脂、既往脑卒中病史、吸烟史、冠状动脉粥样硬化性心脏病；②实验室检查：血常规、血糖、肿瘤标志物、凝血四项[活化部分凝血活酶时间（activated partial thromboplastin time, APTT）、凝血酶原时间（prothrombin time, PT）、纤维蛋白原（fibrinogen, FIB）和凝血酶时间（thrombin time, TT）]和D-二聚体；③风湿结缔组织病相关系列、抗心磷脂抗体；④是否合并其他血栓栓塞事件：如DVT和PE等。

### 1.3 影像学资料

所有患者均行胸部计算机断层扫描（computed tomography, CT）平扫+增强检查、头部MRI平扫+增强检查（排除颅内转移）、头部磁共振血管成像（MR angiography, MRA）、颈部血管彩超、腹部彩超、心电图和心脏彩超。头颅MRI检查均在患者出现急性脑梗死症状的24 h内完成。头颅MRI检查采用Siemens Prisma 3.0 T MRI扫描仪。扫描序列：轴位T1WI/增强T1WI：TE/TR=7/1, 300 ms；T2WI：TE/TR=88/3, 690 ms；DWI（*b*=0 s/mm^2^-1, 000 s/mm^2^）：TE/TR=60/4, 000 ms；所有序列层厚均为5 mm，层间隔0.5 mm。

### 1.4 头部MRI图像分析

由两位医学影像科主治医师共同阅片，分析DWI序列上急性脑梗死灶的部位、累及的血管数量、大小和体积：①部位：大脑半球——额叶、颞叶、顶叶、岛叶、枕叶；深部核团——基底节、丘脑；幕下——脑干、小脑半球；②累及的血管数量：将颅内动脉分为21支供血区，包括双侧大脑前动脉皮质支、双侧大脑前动脉中央支、双侧大脑中动脉皮质支、双侧大脑中动脉中央支、双侧脉络膜前动脉、双侧大脑后动脉皮质支、双侧大脑后动脉中央支、基底动脉、双侧小脑后下动脉、双侧小脑前下动脉及双侧小脑上动脉。记录梗塞灶累及颅内动脉的数量；③数量：分为单发和多发，多发者为非连续性病灶数量在2个或2个以上；④体积：在每一层，手工勾勒梗死灶边缘，得出其面积，将面积乘以层距（层厚5 mm+层间隔0.5 mm），算出每一层的体积，再逐层相加，得出每个梗塞灶的体积；最后算出每例患者所有梗塞灶的总体积。此外，记录头部MRA图像上颅内动脉管腔狭窄的部位及程度。管腔狭窄范围 < 30%、≥30%且≤70%、 > 70%分别定义为轻度、中度及重度狭窄。

### 1.5 D-二聚体水平与急性脑梗死灶累及血管供血区数量的关系

D-二聚体水平的测量与头部MRI平扫检查间隔时间小于24 h。分析所有患者的D-二聚体水平与急性脑梗死病灶累及血管供血区数量的相关性。由于D-二聚体水平和急性脑梗死灶累及血管供血区数量均不符合正态分布，同时也需要考虑4例合并DVT患者和2例合并PE患者对结果的影响。

### 1.6 统计学分析

采用SPSS 22.0统计学软件分析数据，相关性分析采用*Spearman*秩相关分析，*P* < 0.05为差异有统计学意义。

## 2 结果

### 2.1 临床特征

① 临床特点：22例患者在肺癌确诊后6个月-4年后发现TS相关的急性脑梗死；3例患者以急性脑梗死为首发临床表现；②肺癌的病理和临床分期：腺癌22例、鳞癌2例和大细胞癌1例；根据2017年国际抗癌联盟第八版制定的分期标准分期如下：腺癌Ⅱ期2例，Ⅲ期9例，Ⅳ期11例，鳞癌Ⅲ期2例，大细胞癌Ⅳ期1例；③20例患者具有动脉粥样硬化危险因素，包括高血压10例、糖尿病5例、高血脂5例、吸烟10例；④实验室检查：5例患者轻度贫血（Hb > 90 g/L），5例患者中度贫血（Hb < 60 g/L）；5例糖尿病患者服药后空腹血糖水平正常或接近正常。所有患者癌胚抗原（carcinoembryonic antigen, CEA）和糖类抗原125（carbohydrate antigen 125, CA125）均有不同程度升高，2例鳞癌患者鳞状细胞癌抗原（squamous cell carcinoma, SCC）和细胞角蛋白19片段（cytokeratin 19 fragment antigen, CYFRA21-1）升高。所有患者均表现为血小板计数不同程度的增高（320×10^9^个/L-621×10^9^个/L，中位数：425×10^9^个/L；正常值：100×10^9^个/L-300×10^9^个/L），D-二聚体明显升高（2, 800 ng/mL-9, 000 ng/mL，中位数：6, 058 ng/mL；正常值< 300 ng/mL），APTT及PT均有不同程度缩短，FIB正常或接近正常；⑤风湿结缔组织病相关系列及抗心磷脂抗体均为阴性；⑥4例患者合并DVT，2例患者合并PE。

### 2.2 DWI图像特点

① 所有患者均出现急性多发脑梗死，于DWI上表现为明显高信号，ADC值明显减低；②脑梗死部位：大脑半球25例、深部核团11例、幕下7例；③分布：25例均表现为多个血管分布区，累及前后循环20例，仅累及前循环5例；累及的血管供血区数量为5支-13支，平均9支。④数量：0例 < 5处、11例≥5处且 < 10处、14例≥10处；⑤体积：梗塞灶的最大直径从0.2 cm-7 cm，同一患者梗塞灶的大小分布具有随机性；体积范围50.43 cm^3^-201.19 cm^3^。见表 1、图 2及图 3。

**表 1:** NSCLC合并Trousseau综合征以急性脑梗死为表现患者的临床及影像学特征 The clinical and imaging features of acute cerebral infarction in NSCLC patients with Trousseau syndrome

No.	Sex	Age (yr)	Pathological type	Stage	D-dimer (ng/mL)	Features of infarction foci (DWI)			
						Location	Number of involved vessels	Number of infarction foci	Volume (cm^3^)
1	M	78	LUAD	Ⅱ	3, 450	Cerebrum, deep nuclei	7	23	76.12
2	M	43	LUAD	Ⅲ	3, 960	Cerebrum, deep nuclei	8	18	80.99
3	F	58	LUAD	Ⅲ	3, 320	Cerebrum, deep nuclei	8	20	201.19
4	M	42	LUAD	Ⅲ	9, 000	Cerebrum	13	17	129.74
5	M	50	LUAD	Ⅲ	4, 500	Cerebrum, infratentorial regions	10	5	89.37
6	M	57	LUAD	Ⅳ	2, 800	Cerebrum	5	9	175.41
7	F	67	LUAD	Ⅳ	5, 600	Cerebrum	7	8	195.43
8	F	62	LUAD	Ⅲ	4, 560	Cerebrum	7	6	109.69
9	F	65	LUAD	Ⅳ	7, 640	Cerebrum, infratentorial regions, deep nuclei	12	9	139.98
10	F	68	LUAD	Ⅳ	4, 280	Cerebrum, infratentorial regions, deep nuclei	10	9	79.56
11	M	58	LUAD	Ⅳ	3, 520	Cerebrum, deep nuclei	7	10	144.65
12	M	56	LUAD	Ⅱ	7, 190	Cerebrum, deep nuclei	7	15	135.22
13	M	72	LUAD	Ⅳ	7, 650	Cerebrum	7	17	65.35
14	F	40	LUAD	Ⅳ	6, 520	Cerebrum	10	32	82.63
15	M	47	LUAD	Ⅲ	6, 500	Cerebrum, infratentorial regions	9	22	74.49
16	M	50	LUAD	Ⅲ	7, 690	Cerebrum	9	23	123.37
17	M	76	LUAD	Ⅳ	5, 690	Cerebrum	11	7	72.46
18	M	52	LUAD	Ⅳ	6, 800	Cerebrum	11	7	85.09
19	M	68	LUAD	Ⅲ	6, 830	Cerebrum, infratentorial regions	10	8	50.43
20	M	49	LUAD	Ⅳ	6, 230	Cerebrum, infratentorial regions, deep nuclei	11	8	88.66
21	M	57	LUAD	Ⅳ	8, 700	Cerebrum, infratentorial regions, deep nuclei	11	34	137.55
22	M	39	LUAD	Ⅲ	8, 120	Cerebrum, deep nuclei	8	6	140.52
23	M	47	LUSC	Ⅲ	7, 500	Cerebrum, deep nuclei	10	25	77.54
24	M	51	LUSC	Ⅲ	8, 890	Cerebrum	10	28	189.89
25	M	55	LCC	Ⅳ	4, 510	Cerebrum	8	30	145.02
LUAD: Lung adenocarcinoma; LUSC: Lung squamous cell carcinoma; LCC: large cell carcinoma; M: Male; F: Female.									

**图 2:**
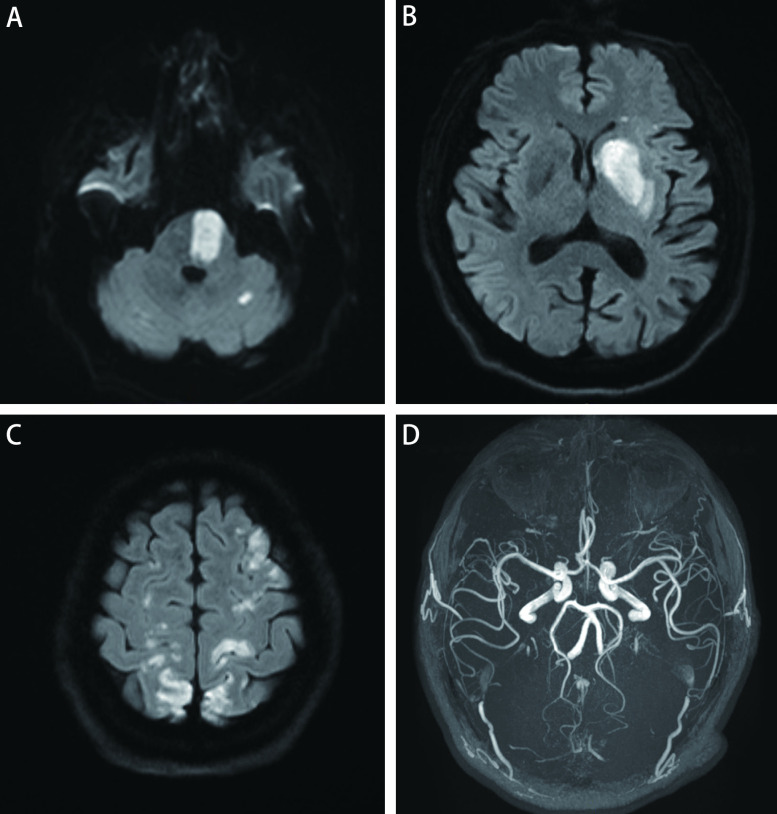
病例9的DWI及MRA图像特征。A-C：双侧额叶、顶叶、左侧基底节区、脑桥及左侧小脑半球可见多发高信号，提示梗死；D：颅内动脉管腔未见明显狭窄。 DWI and MRA feature of case 9. A-C: DWI shows multiple foci of high signal intensity in bilateral frontal lobe, parietal lobe, left basal ganglia, pons and left cerebellar hemisphere, indicating infarction; D: MRA shows no significant narrowing of all the intracranial arterial lumen. DWI: diffusion-weighted imaging; MRA: magnetic resonance angiography.

**图 3:**
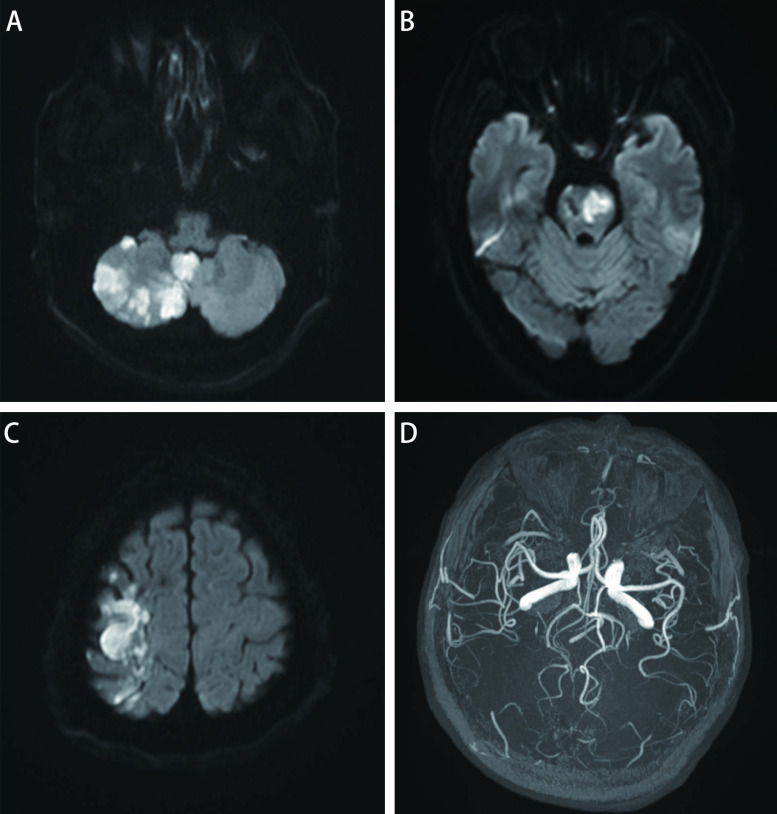
病例19的DWI及MRA图像特征。A-C：右侧额叶、顶叶、脑桥、右侧小脑半球及扁桃体可见多发高信号，提示梗死；D：颅内动脉管腔未见明显狭窄 DWI and MRA feature of case 19. A-C: DWI shows multiple foci of high signal intensity in right frontal lobe, parietal lobe, pons, right cerebellar hemisphere and tonsil, indicating infarction; D: MRA shows no significant narrowing of all the intracranial arterial lumen.

### 2.3 MRA图像分析

25例患者中仅有5例急性脑梗死灶所对应的颅内动脉管腔轻度狭窄。10例患者颅内动脉管腔轻中度狭窄，但均不是梗死灶的责任血管。见图 2及图 3。

### 2.4 D-二聚体水平与急性脑梗死病灶累及血管区域数量的关系

D-二聚体水平与梗死灶累及血管区域的数量呈显著正相关性（*r*=0.589, *P*=0.002）。见图 4。

**图 4:**
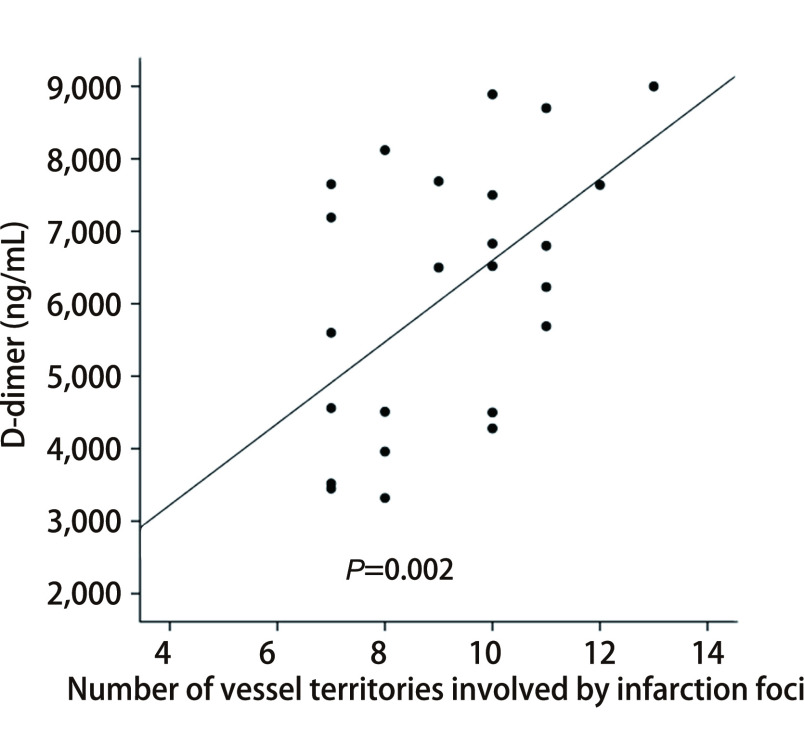
D-二聚体水平与梗死灶累及血管区域数量的相关性 Correlation between D-dimer and the number of vessel territories involved by infarction

## 3 讨论

本研究通过分析NSCLC患者合并TS急性脑梗死灶的影像学特点，发现梗死灶的数目多，累及血管供血区范围广，且血浆D-二聚体水平与梗死灶累及颅内大血管的数量呈正相关。多数恶性肿瘤在其发病过程中均存在不同程度的凝血和纤溶系统的异常，临床上可以从无症状的高凝状态进展为血栓栓塞事件，最终发展为致命性DIC^[4]^。TS的本质可能是患者在凝血和纤溶系统上对肿瘤细胞的防御反应体现，反应适当可限制肿瘤的扩散，反应过强不仅导致血栓栓塞等并发症，还促进肿瘤细胞增殖、侵袭转移及肿瘤组织血管形成。目前认为恶性肿瘤细胞可以通过释放自身生成的促凝因子或刺激其他细胞（内皮细胞、单核-巨噬细胞、血小板等）的促凝活性，激活凝血和纤溶系统^[5]^；此外，手术、放化疗、中心静脉置管等均对血管内皮细胞有不同程度的损伤；晚期恶性肿瘤患者长期卧床、活动减少等均可导致血液流速减慢，导致血液黏滞度增加^[6]^。本研究中患者的D-二聚体中位浓度为6, 058 ng/mL，尽管没有达到DIC的标准，但与传统意义上的血栓性疾病比较，其水平明显升高，我们的结果与Sorensen等^[7, 8]^的报道类似。

本研究中25例NSCLC中有22例为腺癌，和其他研究报道多数TS患者的原发肿瘤为腺癌一致，机理可能是腺癌细胞产生的组织蛋白酶激活机体的凝血系统，分泌的黏蛋白可引起机体的变态反应，使血管内膜或心脏瓣膜的胶原及周围组织退行性变、纤维素样变和上皮细胞脱落，使该部位容易形成血栓或赘生物。此外，Dearborn等^[9]^报道来自胰腺、肺、结肠、乳腺、前列腺和卵巢的腺癌分泌的黏蛋白入血液中可促进黏性和高凝状态；同时黏蛋白还可以与内皮细胞、血小板和淋巴细胞上的某些细胞黏附分子相互作用，最终诱导形成富含血小板的凝血酶。目前的研究^[1, 10]^认为，恶性肿瘤患者合并TS致急性脑梗死灶的致病机制主要是恶性肿瘤引起的血液高凝状态及非细菌性血栓性心内膜炎。血液高凝状态容易出现颅内动脉微小血栓形成和DIC，且颅内原位微小血栓无脑血管区域差异；此外，非细菌性血栓性心内膜炎的赘生物小且容易脱落，故微栓子易随着血液循环而栓塞颅内动脉小分支，上述两种因素导致TS患者急性梗塞灶数目多，累及血管区域更广，我们的研究结果与之前的报道^[8, 11-14]^一致。此外，我们还发现血浆D-二聚体水平与梗死灶累及颅内大血管的数量呈正相关，间接提示NSCLC患者的高凝状态是导致多发急性脑梗死的主要机制。

TS患者合并急性脑梗死需要和其他原因（大动脉粥样硬化、心源性、低灌注和小动脉闭塞性）所致的急性脑梗死鉴别，尽管均为缺血性脑卒中，治疗原则却有所差异。本研究中患者颈动脉超声均未发现颈动脉管腔狭窄超过30%，头部MRA显示梗塞区域的对应的责任动脉管腔狭窄均未超过30%，不符合大动脉粥样硬化性缺血性脑卒中的诊断。所有患者心脏彩超及心电图均未发现有可能形成心脏栓子的疾病，基本排除心源性脑栓塞。患者均无低血压、无引起心输出量明显减少的疾病、无重度贫血，梗塞灶不符合“分水岭”分布，不符合低灌注所致的脑梗塞。小动脉闭塞所致的梗塞多为腔隙性梗塞，最大直径一般 < 1.5 cm，多位于深穿支动脉供血的脑区，如半卵圆中心、基底节-丘脑及脑干等。本研究中的梗塞灶分布具有随机性，皮质区和深部脑区均同时受累，不符合小动脉闭塞性脑卒中。此外，TS患者合并急性脑梗死需要与脑转移瘤鉴别，且两者可以并存，但转移瘤多伴有瘤周水肿，增强检查后瘤灶呈环形或结节状强化，两者容易鉴别。

TS往往发生在晚期恶性肿瘤患者中，但少见情况下可能是恶性肿瘤的首发临床表现^[15]^。本组25例患者中22例在肺癌确诊后6个月-4年后发现TS相关的急性脑梗死；3例患者以急性脑梗死为首发临床表现。对于患者出现急性多发性脑梗死灶，且病灶累及多个动脉供血区，D-二聚体水平显著升高，需要警惕TS存在的可能性。肿瘤标志物的检测及全面的影像学检查可排除潜在的恶性肿瘤，尤其是肺或消化系统来源的腺癌，以期尽早发现肿瘤以及应用抗凝治疗。

本研究为回顾性研究，样本量小，不能完全排除患者可能同时存在其他原因所致的急性脑梗死，未来需要大样本的前瞻性队列研究来进一步明确其临床和影像学特征。NSCLC合并TS导致急性脑梗死病灶常多发，且累及多个动脉供血区，伴有血浆D-二聚体显著升高。对于无明显的传统缺血性脑卒中危险因素、无头颈部动脉管腔中重度狭窄、无可能形成心脏栓子疾病的患者，而影像学表现为急性多发性脑梗死且累及多个动脉供血区，伴有D-二聚体显著升高，需要考虑到TS的可能性，建议筛查隐匿性肿瘤。
